# Relational Differential Dynamic Logic

**DOI:** 10.1007/978-3-030-45190-5_11

**Published:** 2020-03-13

**Authors:** Juraj Kolčák, Jérémy Dubut, Ichiro Hasuo, Shin-ya Katsumata, David Sprunger, Akihisa Yamada

**Affiliations:** 8 Johannes Kepler University, Linz, Austria; 9 University of Birmingham, Birmingham, UK; 10 LSV, CNRS & ENS Paris-Saclay, Université Paris-Saclay, Cachan, France; 11 National Institute of Informatics, Tokyo, Japan; 12 Japanese-French Laboratory for Informatics, CNRS IRL 3527, Tokyo, Japan; 13 The Graduate University for Advanced Studies (SOKENDAI), Tokyo, Japan

**Keywords:** hybrid system, cyber-physical system, formal verification, theorem proving, dynamic logic

## Abstract

In the field of quality assurance of hybrid systems, Platzer’s *differential dynamic logic* (dL) is widely recognized as a deductive verification method with solid mathematical foundations and sophisticated tool support. Motivated by case studies provided by our industry partner, we study a *relational extension* of dL, aiming to formally prove statements such as “an earlier engagement of the emergency brake yields a smaller collision speed.” A main technical challenge is to combine two dynamics, so that the powerful inference rules of dL (such as the differential invariant rules) can be applied to such relational reasoning, yet in such a way that we relate two different time points. Our contributions are a semantical theory of *time stretching*, and the resulting *synchronization* rule that expresses time stretching by the syntactic operation of Lie derivative. We implemented this rule as an extension of KeYmaera X, by which we successfully verified relational properties of a few models taken from the automotive domain.

### 1 Introduction

**Hybrid Systems** *Cyber-physical systems* (CPSs) have been studied as a subject in their own right for over a decade, but the rise of *automated driving* in the last few years has created a panoply of challenges in the quality assurance of these systems. In the foreseeable future, millions of cars will be driving on streets

* Thanks are due to Stefan Mitsch, André Platzer, and Yong Kiam Tan for useful tips on the KeYmaera X source code; and to Kenji Kamijo, Yoshiyuki Shinya, and Takamasa Suetomi from Mazda Motor Corporation for helpful discussions. The authors are supported by ERATO HASUO Metamathematics for Systems Design Project (No. JPMJER1603), JST. I.H. is supported by Grant-in-Aid No. 15KT0012, JSPS. J.D. is supported by Grant-in-aid No. 19K20215, JSPS. The work was done during J.K.'s internship at the National Institute of Informatics, Tokyo, Japan.

with unprecedented degrees of automation; ensuring the safety and reliability of these automated driving systems is a pressing social and economic challenge.

The *hybridity* of cyber-physical systems, the combination of continuous physical dynamics and discrete digital control, poses unique scientific challenges. To address these challenges, two communities have naturally joined forces: *control theory* whose traditional application domain is continuous dynamics and *formal methods* that have mainly focused on the analysis of software systems. This has been a fruitful cross-pollination: techniques from formal methods such as bisimilarity [9] and temporal logic specification [8] have been imported to control theory, and conversely, control theory notions such as Lyapunov functions have been used in formal methods [26].

**Deductive Verification of Hybrid Systems** In the formal methods community, two major classes of techniques are *model checking* (usually automata-based and automatic) and *deductive verification* (based on logic and can be automated or interactive). Model checking techniques rely on exhaustive search in state spaces and therefore cannot be applied *per se* to hybrid systems with infinite state spaces. This has led to the active study of *discrete abstraction* of hybrid dynamics, see e.g. [9]; or of *bounded model checking*, see [5].

In contrast, nothing immediately rules out the use of the deductive approach for hybrid systems. Finitely many variables in logical formulas can represent infinitely many states, and proofs in suitably designed logics are valid even when the semantic domain is uncountable. That said, designing such a logic, proving the soundness of its rules, and showing that logics is actually *useful* in hybrid system verification is a difficult task.

Platzer's *differential dynamic logic* dL [21] is a remarkable success in this direction. Its syntax is systematic and intuitive, extending the classic formalism of *dynamic logic* [10] with differential equations as programs. Its proof rules encapsulate several essential proof principles about differential equations, including a *differential invariant* (DI) rule for universal properties and *side deduction* for existential properties. The logic dL has served as a general platform that accommodates a variety of techniques, including those which come from real algebraic geometry [22]. Furthermore, dL comes with sophisticated tool support: the latest tool KeYmaera X [15] comes with graphical interface for interactive proving and a number of automation heuristics.

**Relational Reasoning on Hybrid Systems** In this work, we introduce proof-based techniques for *relational reasoning* to the deductive verification of hybrid systems. Here, by relational reasoning we mean analyzing how changes in the system will affect the overall system behavior. One of the applications of such reasoning in our mind is to deduce the safety of a system by checking the most aggressive settings. To make such reduction sound, we need to verify that less aggressive versions result in less dangerous outcomes than the aggressive ones. As a simple example, consider the following case distilled from our collaboration with an industrial partner.

**Example 1 (leading example: collision speed).** Consider two cars $\overline{\mathrm{C}}$ and $\underline{\mathrm{C}}$, whose positions and velocities are real numbers denoted by $\overline{x}, \underline{x}$ and $\overline{v}, \underline{v}$,

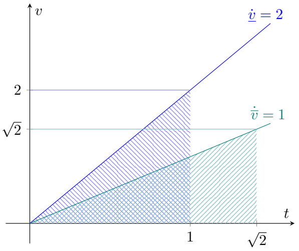

The two hatched areas designate the traveled distances ($\overline{x} = \underline{x} = 1$). We can compute the collision speeds ($\overline{v} = \sqrt{2}$ and $\underline{v} = 2$) via the closed-form solutions of the differential equations (1), concluding $\overline{v} \leq \underline{v}$ when $\overline{x} = \underline{x} = 1$.

**Fig. 1.** An ad-hoc proof for Example 1

respectively. Their dynamics are governed by the following differential equations:

$$\dot{\overline{x}} = \overline{v}, \quad \dot{\overline{v}} = 1; \qquad \dot{\underline{x}} = \underline{v}, \quad \dot{\underline{v}} = 2. \tag{1}$$

Both cars start at the same position at rest ($\overline{x} = \underline{x} = 0 \wedge \overline{v} = \underline{v} = 0$), and both drive towards a wall at position 1. We consider this question: *which car is traveling faster when it hits the wall?*

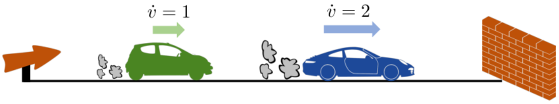

The second car, $\underline{\mathrm{C}}$, has strictly greater acceleration all the time, so we can imagine that $\underline{\mathrm{C}}$ hits the wall harder. This hypothesis turns out to be correct, but we are more interested in how this claim could be proven.

A simple proof would be to solve the differential equation exactly and notice $\underline{\mathrm{C}}$ has greater velocity at the end of its run. However, it is known that closed-form solutions are scarce for ODEs—we want a proof method that is more general.

Another possible argument is based on the relationship between the accelerations. Since the second car's acceleration is greater at every point in time, we might be tempted to conclude that the second car's velocity must always be greater than the first car's, based on the monotonicity of integration: $\overline{a}(t) \leq \underline{a}(t) \Rightarrow \overline{v}(t) = \int_0^T \overline{a}(t)\,\mathrm{d}t \leq \int_0^T \underline{a}(t)\,\mathrm{d}t = \underline{v}(t)$. However, this reasoning has a flaw. $\underline{\mathrm{C}}$ reaches the wall at an earlier point in time than $\overline{\mathrm{C}}$, and therefore $\overline{\mathrm{C}}$ has more time to accelerate. In the end, we have to compare $\int_0^{\overline{T}} \overline{a}(t)\,\mathrm{d}t$ and $\int_0^{\underline{T}} \underline{a}(t)\,\mathrm{d}t$ where $\overline{a}(t) \leq \underline{a}(t)$ for all $t \in [0, \underline{T}]$ but $\overline{T} > \underline{T}$, as depicted in figure 1.

Our solution, roughly stated, is to compare the two cars at the same points *in space* by reparametrizing time for one of the two cars. This parametrization is specially chosen to ensure the two cars pass through the same points in space at the same points in time.

Our current work is about a logical infrastructure needed to support this kind of relational reasoning comparing two different dynamics, based on $\mathsf{dL}$. Our semantical theory, as well as the resulting syntactic extension of $\mathsf{dL}$ by what we call the synchronization rule, generalizes the kind of reasoning in Example 1 using the notion of *time stretching*.

**Technical Contributions** We make the following technical contributions.

1. **Formulation of relational reasoning in dL**. We find that relational properties are expressible in dL, using disjoint variables in a sequential composition. This representation, however, does not allow the use of the rich logical infrastructure of dL (such as the (DI) rule).
2. **Time stretching, semantically and syntactically**. To alleviate this difficulty, we first develop the theory of *time stretching*, so that we can compare two dynamics at different timepoints (cf. Example 1). Accommodating this semantical notion in dL and KeYmaera X is not possible *per se*. We introduce an indirect syntactic alternative, which turns out to be better suited in fact to many case studies (where we compare the two dynamics at the same "position," much like in Example 1). The resulting *synchronization* rule in dL has a clean presentation (Theorem 24), owing to the syntactic Lie derivative operator in dL.
3. **Implementation and case studies**. We implemented the new synchronization rule as an extension of KeYmaera X. We used it successfully for establishing nontrivial relational properties in case studies taken from the automotive domain.

**Relational Reasoning in Practice** We contend relational reasoning has practical significance based on our collaboration with an industry partner. Relational properties, especially with an aspect of *monotonicity*, abound in real-world examples. In particular, we have often encountered situations where we have a parametrized model $M(p)$ and need to show a property of the form:

$$p_1 < p_2 \text{ implies } M(p_2) \text{ is less safe than } M(p_1). \tag{2}$$

These properties occur especially in the context of *product lines*, where the same model can come in many slight variants. Example 1 is such a situation.

Relational statements (such as monotonicity) are easy to state and interpret. Intuitions about the *direction of* the change in a behavior of a system resulting from the change of a parameter are more often valid than intuitions about the *amount of* such a change. These kinds of simple statements are often used by engineers to establish the basic credibility of a model. Qualitative, relational properties also tend to be easier to prove than exact, quantitative properties.

Finally, monotonicity can serve as a powerful technique in *test-case reduction*. If a safety property is too complex to be deductively verified, one usually turns to testing. It is often still possible to establish a simple monotonicity property of the form (2). This can powerfully boost testing efforts: one can focus exclusively on establishing safety for the extreme case $M(p_{\max})$.

**Related Work** Since this work is about its relational extension, the works we mentioned on dL are naturally relevant. We discuss other related works here.

Simulink (Mathworks, Inc.) is an industry standard in modeling hybrid systems, but unfortunately Simulink models do not come with rigorously defined semantics. Therefore, while integration with Simulink is highly desirable any quality assurance methods for hybrid systems, formal verification methods require some work to set up the semantics for Simulink models. The recent work [12] tackles this problem, identifying a fragment of Simulink, and devising a translator from Simulink models to dL programs. Their translation is ingenious, and their tool is capable of proving rather complicated properties when used in combination with KeYmaera X [15].

Relational extensions of the *Floyd–Hoare logic*—which can be thought of as a discrete-time version of dL—have been energetically pursued especially in the context of *differential privacy* [4,2,3].

In deductive verification of hybrid systems, an approach alternative to dL uses *nonstandard analysis* [23] and regards continuous dynamics as if they were discrete due to the existence of infinitesimal elements [24,25]. The logic used in that framework is exactly the same as the classic Floyd–Hoare logic, and the soundness of the logic in the hybrid setting is shown by a model-theoretic result called the *transfer principle*. Its tool support has been pursued as well [11].

This is not the first time that relational reasoning—in a general sense—has been pursued in dL. Specifically, Loos and Platzer introduce the *refinement* primitive $\beta \leq \alpha$, which asserts a refinement relation between two hybrid dynamics, meaning the set of successor states of $\beta$ is included in that of $\alpha$ [14]. This kind of relation is inspired by the software engineering paradigm of incremental modeling (supported by languages and tools such as Event-B [1,6]); the result is a rigorous deductive framework for refining an abstract model (with more nondeterminism) into a more concrete one (with less nondeterminism). In contrast, we compare one concrete model (not necessarily with nondeterminism) with another. Thus, our notion of relational reasoning builds more on relational extensions of the Floyd–Hoare logic [4,2,3] than on Event-B. Combining these two orthogonal kinds of relational extensions of dL is important future work.

**Organization** In Section 2, we recall some basics of differential dynamic logic dL: its syntax, semantics and some proof rules. Our main goal, relational reasoning, is formulated in Section 3, where we identify difficulties in doing so in the original dL. In Section 4 we introduce the semantical notion of time stretching, and turn its theory into the new synchronization rule. After introducing our implementation in Section 5, we describe our three case studies in Section 6.

The appendix containing omitted proofs and details, the source code and the artifact are found at http://group-mmm.org/rddl_tacas_2020/.

### 2 Preliminaries: Syntax and Semantics of the Logic dL

We recall some of the basics of *differential dynamic logic* (dL). The interested reader is referred to [19,20] for full details.

**Definition 2 (language).** We fix a set $\mathcal{V}$ of *variables*, denoted by $x, y, \ldots$. The set of *terms* is defined by the following grammar:

$$e, f, g, \ldots \ ::= \ x \mid n \mid -e \mid e + f \mid e \cdot f \mid e/f$$

where $x \in \mathcal{V}$ and $n \in \mathbb{N}$. First-order *formulas* are defined by

$$P, Q, \ldots \ ::= \ e \leq f \mid \neg P \mid P \wedge Q \mid \forall x. P$$

A *state* is a function mapping each variable to a real number, $\omega : \mathcal{V} \to \mathbb{R}$. We denote the set of all states by $\mathbb{R}^{\mathcal{V}}$. Given a state, each term has a valuation in the reals, and each formula has a valuation in Booleans defined by the usual induction. We denote these by $[\![e]\!]_\omega \in \mathbb{R}$ and $[\![P]\!]_\omega \in \{\text{TRUE}, \text{FALSE}\}$, respectively. The *models* of a first-order formula $P$ are the states satisfying $P$, $[\![P]\!] := \{\omega \in \mathbb{R}^{\mathcal{V}} \mid [\![P]\!]_\omega = \text{TRUE}\}$.

We use classical shorthands, including $e = f := e \leq f \wedge f \leq e$, $P \vee Q := \neg(\neg P \wedge \neg Q)$, $\exists x. P := \neg(\forall x. \neg P)$, and $\top := 0 \leq 0$. We denote a vector $(e_1, \ldots, e_n)$ of terms (or variables) by $\mathbf{e}$ when the length $n$ is irrelevant or clear from the context.

We now introduce the syntax of hybrid programs.

**Definition 3 (hybrid programs).** The set $\mathcal{HP}(\mathcal{V})$ of *hybrid programs* over variables $\mathcal{V}$ is given by the following grammar:

$$\alpha_1, \alpha_2, \ldots \ ::= \ ?P \ \mid \ x := e \mid \dot{x}_1 = e_1, \ldots, \dot{x}_n = e_n \,\&\, Q \ \mid \ \alpha_1; \alpha_2 \ \mid \ \alpha_1 \cup \alpha_2 \mid \alpha_1^*$$

We may also abbreviate $\dot{x}_1 = e_1, \ldots, \dot{x}_n = e_n$ by $\dot{\mathbf{x}} = \mathbf{e}$. Hybrid programs of the form $\dot{\mathbf{x}} = \mathbf{e} \,\&\, Q$ are especially important in this work. We call such a program *differential dynamics*, where $\dot{\mathbf{x}} = \mathbf{e}$ is its *differential equation* and the first-order formula $Q$ is its *evolution domain constraint*. The intuitive meaning of such a program is that the values of the variables $\mathbf{x}$ evolve continuously in time according to $\dot{\mathbf{x}} = \mathbf{e}$, as long as $Q$ is satisfied at the current value of $\mathbf{x}$. If we see differential dynamics as a continuous analog of loops, then $Q$ plays the role of guard and $\dot{\mathbf{x}} = \mathbf{e}$ plays the role of body.[5] We write $\dot{\mathbf{x}} = \mathbf{e}$ instead of $\dot{\mathbf{x}} = \mathbf{e} \,\&\, \top$.

**Definition 4 (solutions).** A mapping $\psi : [0, T) \to \mathbb{R}^{\mathcal{V}}$ with $T \in [0, \infty]$ is called a *solution* of a differential equation $\dot{x}_1 = e_1, \ldots, \dot{x}_n = e_n$ if $\psi$ is differentiable in $[0, T)$ and, whenever $t \in [0, T)$, $\dot{\psi}(t)(x_i) = [\![e_i]\!]_{\psi(t)}$ for $i \in \{1, \ldots, n\}$ and $\dot{\psi}(t)(y) = 0$ for any $y \in \mathcal{V} \setminus \{x_1, \ldots, x_n\}$.

According to the Picard–Lindelöf theorem [13], for each differential equation $\dot{\mathbf{x}} = \mathbf{e}$ and each state $\omega$, there is a unique maximal solution $\psi_\omega : [0, T_\omega) \to \mathbb{R}^{\mathcal{V}}$ of the differential equation satisfying $\psi_\omega(0) = \omega$.

**Definition 5 (semantics of hybrid programs).** The *semantics* of a hybrid program $\alpha$ is a relation $\dashv\![\![\alpha]\!]\!\mapsto \subseteq \mathbb{R}^{\mathcal{V}} \times \mathbb{R}^{\mathcal{V}}$ on states, defined by:

[5] This analogy is not perfect: a typical while loop can only exit when its guard is false, whereas a hybrid program can exit the differential dynamics while $Q$ is satisfied.

1. $\llbracket ?P \rrbracket = \{(\omega, \omega) \mid \omega \in \llbracket P \rrbracket\}$,
2. $\llbracket x := e \rrbracket = \{(\omega, \omega') \mid \omega'(x) = \llbracket e \rrbracket_\omega$ and $\omega'(y) = \omega(y)$ for all $y \neq x\}$,
3. $\llbracket \dot{\mathbf{x}} = \mathbf{e} \,\&\, Q \rrbracket = \{(\omega, \psi_\omega(t)) \mid \omega \in \mathbb{R}^{\mathcal{V}},\ t \in [0, T_\omega), \psi_\omega([0,t]) \subseteq \llbracket Q \rrbracket\}$,
4. $\llbracket \alpha_1 \cup \alpha_2 \rrbracket = \llbracket \alpha_1 \rrbracket \cup \llbracket \alpha_2 \rrbracket$,
5. $\llbracket \alpha_1 ; \alpha_2 \rrbracket = \llbracket \alpha_1 \rrbracket ; \llbracket \alpha_2 \rrbracket$ where ; denotes relation composition, and
6. $\llbracket \alpha^* \rrbracket = (\llbracket \alpha \rrbracket)^*$ where $^*$ denotes the reflexive transitive closure.

**Definition 6 (dL formulas).** *Modal formulas* extend first-order formulas and are defined by the following grammar:

$$\varphi, \varphi_1, \varphi_2, \dots \ ::= e \leq f \mid \neg\varphi \mid \varphi_1 \wedge \varphi_2 \mid \forall x.\, \varphi \mid [\alpha]\varphi.$$

As usual, we write $\langle\alpha\rangle\varphi$ to abbreviate $\neg[\alpha]\neg\varphi$. We will also call modal formulas "dL formulas" since these are the widest class of formulas in dL.

The Boolean valuation $\llbracket \varphi \rrbracket_\omega$ of a modal formula $\varphi$ in a state $\omega$ is defined in the same way as for first-order formulas, with the addition of $\llbracket [\alpha]\varphi \rrbracket_\omega = \text{TRUE}$ if and only if $\llbracket \varphi \rrbracket_{\omega'} = \text{TRUE}$ for all $\omega'$ such that $\omega \llbracket \alpha \rrbracket \omega'$.

We take the sequent-calculus style proof system for dL, following [22]. It has judgments of the form $\Gamma \vdash \varphi$, where $\Gamma$ is a set of modal formulas and $\varphi$ is a single modal formula. One of the most fundamental axiom is

$$[\dot{\mathbf{x}} = \mathbf{e} \,\&\, Q]\phi \iff \forall t \geq 0.\, (\forall v \in [0, u].\ [x := f(v)]Q) \Rightarrow [x := f(u)]\phi \quad \text{(solve)}$$

where $f(t)$ is a term with a fresh variable $t$ such that $\llbracket f \rrbracket$ is a solution of $\dot{\mathbf{x}} = \mathbf{e}$ and $\llbracket f(0) \rrbracket = \mathsf{id}$.

Some other rules of dL, such as the differential invariant rule (DI) that is central in many proofs, are introduced later in Definition 13.

### 3 Relational Differential Dynamic Logic

Intuitively, we want a way to describe two dynamics that are executed in parallel, and compare their outputs. In terms of (nondeterministic) transition systems, parallel composition is available via tensor products.

**Definition 7 (tensor product).** Given two transition systems $(S, R)$ and $(S', R')$, their *tensor product* $(S \times S', R \otimes R')$ is defined to be the transition system whose transition relation is given by

$$R \otimes R' := \{(s, s'), (t, t') \mid (s, t) \in R, (s', t') \in R'\}.$$

No extension of the dL syntax is needed to model tensor products: disjointness of the variables of the two systems suffices. From now on we split variables into two disjoint sets: $\mathcal{V} = \overline{\mathcal{V}} \uplus \underline{\mathcal{V}}$. We denote variables in $\overline{\mathcal{V}}$ by $\overline{x}, \overline{y}, \dots$ and those in $\underline{\mathcal{V}}$ by $\underline{x}, \underline{y}, \dots$. Terms in $\mathcal{T}(\overline{\mathcal{V}})$, first-order formulas in $\mathcal{F}ml(\overline{\mathcal{V}})$, and programs in $\mathcal{HP}(\overline{\mathcal{V}})$ are denoted by $\overline{e}, \overline{f}, \dots, \overline{P}, \overline{Q}, \dots$, and $\overline{\alpha}, \overline{\beta}, \dots$, and similarly for the corresponding constructs with $\underline{\mathcal{V}}$.

An easy proof of the following fact can be found in the appendix.

**Proposition 8.** $-\llbracket \overline{\alpha} \rrbracket\!\!\rightarrow \otimes -\llbracket \underline{\alpha} \rrbracket\!\!\rightarrow = -\llbracket \overline{\alpha}; \underline{\alpha} \rrbracket\!\!\rightarrow$ □

Scenarios with two parallel differential dynamics are the main focus of this work. We formalize an assertion relating two dynamics using the following format. It is a syntactic counterpart of Proposition 8.

**Definition 9 (relational differential dynamics).** We call hybrid programs of the following form *relational differential dynamics (RDD)*

$$\dot{\overline{\mathbf{x}}} = \overline{\mathbf{e}} \,\&\, \overline{Q}\;;\quad \dot{\underline{\mathbf{x}}} = \underline{\mathbf{e}} \,\&\, \underline{Q} \tag{3}$$

Now that we have ways to express separate systems evolving in parallel, we turn to the construction of proofs which reason about their relationships.

**Example 10.** Using RDD, the problem in Example 1 is expressed as $\Gamma_C \vdash [\overline{\delta_C}; \underline{\delta_C}]\phi_C$ where $\overline{\delta_C} := (\dot{\overline{x}} = \overline{v}, \dot{\overline{v}} = 1)$, $\underline{\delta_C} := (\dot{\underline{x}} = \underline{v}, \dot{\underline{v}} = 2)$, $\Gamma_C := \{\overline{x} = \underline{x} = 0,\ \overline{v} = \underline{v} = 0\}$ is the precondition, and $\phi_C := (\overline{x} = \underline{x} = 1 \Rightarrow \overline{v} \leq \underline{v})$ is the postcondition.

Let us prove, in KeYmaera X, the RDD sequent $\Gamma_C \vdash [\overline{\delta_C}; \underline{\delta_C}]\phi_C$. In KeYmaera X, the only applicable rule to this sequent turns it into $\Gamma_C \vdash [\overline{\delta_C}][\underline{\delta_C}]\phi_C$. We then explicitly "solve" the second dynamics, yielding the following goal:

$$\Gamma_C \vdash [\overline{\delta_C}]\forall \underline{t} \geq 0.\,(\overline{x} = \underline{x} + \underline{v}\cdot\underline{t} + \underline{t}^2 = 1 \Rightarrow \overline{v} \leq \underline{v} + \underline{t}) \tag{4}$$

where $\underline{x}$ and $\underline{v}$ in $\phi_C$ are replaced by their explicit solutions with respect to the freshly introduced time variable $\underline{t}$. Again differential invariant rules do not apply to (4), so one must solve the first dynamics, too, yielding

$$\Gamma_C \vdash \forall \overline{t} \geq 0.\forall \underline{t} \geq 0.\left(\overline{x} + \overline{v}\cdot\overline{t} + \overline{t}^2/2 = \underline{x} + \underline{v}\cdot\underline{t} + \underline{t}^2 = 1 \Rightarrow \overline{v} + \overline{t} \leq \underline{v} + \underline{t}\right)$$

Since this goal is first order, the quantifier elimination, a central proof technique in KeYmaera X [18], proves the goal.

The above example worked out since it admits explicit solutions expressible in dL. This is not always the case as the following example demonstrates.

**Example 11.** We consider two objects moving through fluids subjected to different kinds of drag. One object moves through a viscous fluid and is therefore subject to linear drag; its dynamics are $\underline{\delta_F} := (\dot{\underline{x}} = \underline{v}, \dot{\underline{v}} = -\underline{v})$.

The other object moves through a less viscous fluid and is subject to turbulent drag; its dynamics are $\overline{\delta_F} := (\dot{\overline{x}} = \overline{v}, \dot{\overline{v}} = -\overline{v}^2)$. Our goal is to show that the latter has higher speed when both objects reach a certain point in space $(\overline{x} = \underline{x} = l)$.

The following functions $\underline{v}^*, \underline{x}^*, \overline{v}^*$ and $\overline{x}^*$ are solutions of the dynamics.

$$\underline{v}^*(\underline{t}) = \underline{v}_0 \cdot e^{-\underline{t}} \qquad \underline{x}^*(\underline{t}) = \underline{x}_0 + \underline{v}_0 \cdot (1 - e^{-\underline{t}})$$

$$\overline{v}^*(\overline{t}) = \frac{\overline{v}_0}{1 + \overline{v}_0 \cdot \overline{t}} \qquad \overline{x}^*(\overline{t}) = \overline{x}_0 + \log(1 + \overline{v}_0 \cdot \overline{t})$$

where $\underline{v}_0$ etc. denote the initial values. Unfortunately, we cannot express exponentiations and logarithms in KeYmaera X, and thus the "solve" rule that we used in Example 10 cannot be applied here.

One obvious solution to this would be to add support for exponentiations and logarithms in KeYmaera X, but this would break the decidability of the underlying first order logic, which is a major feature of dL [18]. In fact, the same issue occurs even in standard use cases of KeYmaera X, and motivated the introduction of proof rules which do not demand explicit solutions to differential dynamics [20,22] using the *Lie derivative*.

**Definition 12 (formal Lie derivative in dL from [20,22]).** The formal Lie derivative of a term $f$ along dynamics $\delta \equiv (\dot{\mathbf{x}} = \mathbf{e} \mathbin{\&} Q)$ of dimension $n$ is a dL term $\mathcal{L}_\delta f \in \mathcal{T}(\mathcal{V})$ given by[6]

$$\mathcal{L}_\delta f := \tfrac{\partial}{\partial x_1} f \cdot e_1 + \cdots + \tfrac{\partial}{\partial x_n} f \cdot e_n$$

**Definition 13 (proof rules from [20,22]).** The following rules are sound:

$$\frac{\Gamma, Q \vdash f \sim 0 \quad \Gamma \vdash [\delta]\mathcal{L}_\delta f \simeq 0}{\Gamma \vdash [\delta] f \sim 0}\ \mathrm{DI} \qquad \frac{\Gamma \vdash p \sim 0 \quad Q \vdash \mathcal{L}_\delta p \simeq g \cdot p}{\Gamma \vdash [\delta] p \sim 0}\ \mathrm{Dbx}$$

where $\delta \equiv (\dot{\mathbf{x}} = \mathbf{e} \mathbin{\&} Q)$, $(\sim, \simeq) \in \{\, (=,=), (>,\geq), (\geq,\geq) \,\}$, and $g$ is any term without division.

The differential invariant rule (DI) is the central rule for proving safety properties [20,22]: it reduces a global property of the dynamics to local reasoning by means of Lie derivatives. The Darboux inequality rule (Dbx) is derived from real algebraic geometry; see e.g. [22].

**Example 14.** Consider an example differential dynamics in one variable, $\dot{x} = 2$. Suppose we want to show that $x \geq 0$ holds after following these dynamics for any amount of time, starting from $x = 1$. One way to do this is to show that (1) this predicate holds initially and (2) the time derivative of $x$ is always nonnegative. These are precisely the two premises of the (DI) rule: to show the sequent $x = 1 \vdash [\dot{x} = 2]x \geq 0$ (DI) requires us to prove (1) $x = 1 \vdash x \geq 0$ and (2) $x = 1 \vdash [\dot{x} = 2]\mathcal{L}_{\dot{x}=2}\, x \geq 0$, where $\mathcal{L}_{\dot{x}=2}\, x = 2$. Note that we give an initial condition $x = 1$ in the precedent of this sequent.

### 4 Synchronizing Dynamics

The intuitive explanation of the RDD construction of Definition 9 is a "serialization" of two dynamics. This construction however does not match the (DI)

[6] It is easy to see that the *derivative* of a term $t \in \mathcal{T}(\mathcal{V})$ with respect to $x \in \mathcal{V}$ can be given as a dL term $\frac{\partial}{\partial x} e \in \mathcal{T}(\mathcal{V})$ such that $[\![ \frac{\partial}{\partial x} e ]\!] = \frac{\partial}{\partial x} [\![ e ]\!]$. The definition of $\frac{\partial}{\partial x} e$ is inductive with respect to the term $e$.

and (Dbx) rules, as they accept only one dynamics followed by a comparison. In order to make use of these rules in our relational reasoning, we introduce another proof method. It "synchronizes" two dynamics.

After some theoretical preparations we define the new rule and prove its soundness. We will illustrate the usefulness of this rule in Section 6, through some case studies that are inspired by our collaboration with the industry.

#### 4.1 Time Stretching

A key theoretical tool towards the soundness of our synchronization rule is called *time stretching*. Its idea is very similar to the technique of *time-reparametrization* for ODEs [7].

**Definition 15 (time stretch function).** Let $T \in \mathbb{R}_{\geq 0}$. A function $K : [0, T] \to \mathbb{R}_{\geq 0}$ is a *time stretch function* if $K(0) = 0$, $K$ is continuously differentiable and $\dot{K}(t) > 0$ for each $t \in [0, T]$.

**Remark 16.** The condition $\dot{K}(t) > 0$ ensures that $K$ is strictly increasing and is a bijection from $[0, T]$ to $[0, K(T)]$. The inverse of $K$ is $K^{-1} : [0, K(T)] \to [0, T]$, and it is straightforward to check $K^{-1}$ is another time stretch function.

The next results tell us how to turn an ODE into another, given a time stretching function $K$, so that a time-stretch $\psi \circ K$ of a solution $\psi$ of one becomes a solution of the other.

**Lemma 17.** *Suppose $f : \mathbb{R}^{\mathcal{V}} \to \mathbb{R}^{\mathcal{V}}$ is a vector field and $K : [0, T] \to [0, K(T)]$ is a time stretch function. If $\psi : [0, K(T)) \to \mathbb{R}^{\mathcal{V}}$ satisfies $\dot{\psi}(s) = f(\psi(s))$ for all $s \in [0, K(T))$, then the function $\rho = \psi \circ K : [0, T) \to \mathbb{R}^{\mathcal{V}}$ satisfies $\dot{\rho}(t) = \dot{K}(t) \cdot f(\rho(t))$ for all $t \in [0, T)$.*

*Proof.* We have $\dot{\rho}(t) = \dot{K}(t) \cdot \dot{\psi}(K(t)) = \dot{K}(t) \cdot f(\psi(K(t))) = \dot{K}(t) \cdot f(\rho(t))$, where the first equality is by the definitions and the chain rule, the second equality is by the assumption on $\dot{\psi}$, and the last equality is by the definition of $\rho$. □

Since the inverse of a time stretch function is another time stretch function, we obtain the following corollary of Lemma 17.

**Corollary 18.** *Let $K : [0, T] \to [0, K(T)]$ be a time stretch function. Let $\rho : [0, T) \to \mathbb{R}^{\mathcal{V}}$ satisfy $\dot{\rho}(t) = \dot{K}(t) \cdot f(\rho(t))$ whenever $0 \leq t < T$. Then the function $\psi : [0, K(T)) \to \mathbb{R}^{\mathcal{V}}$, defined by $\psi(s) := \rho(K^{-1}(s))$, satisfies $\dot{\psi}(s) = f(\psi(s))$ whenever $0 \leq s < K(T)$.* □

#### 4.2 Towards a Syntactic Representation

So far our time-stretch function $K$ has been a semantical object. Here we introduce a syntactic way of reasoning via time-stretch functions. Since a desired time-stretch function is not necessarily expressible in dL, our syntactic reasoning uses an indirect method that exploits a pair of functions called a synchronizer. We will be eventually led to a syntactic reasoning rule (Sync) (Thm. 24).

Given a term $g \in \mathcal{T}(X)$ and a mapping $\psi : [0, T) \to \mathbb{R}^X$, we define $g_\psi : [0, T) \to \mathbb{R}$ by

$$g_\psi(t) := [\![g]\!]_{\psi(t)}. \tag{5}$$

Intuitively, $g_\psi(t)$ is the value of $g$ at time $t$ when we follow the dynamics whose solution is $\psi$.

**Definition 19 (synchronizers).** Let $(\overline{\delta}, \underline{\delta})$ be a pair of dynamics, $(\overline{\omega}, \underline{\omega}) \in \mathbb{R}^{\overline{\mathcal{V}}} \times \mathbb{R}^{\underline{\mathcal{V}}}$ be a pair of states, and $\overline{\psi} : [0, \overline{T}) \to \mathbb{R}^{\overline{\mathcal{V}}}$ and $\underline{\psi} : [0, \underline{T}) \to \mathbb{R}^{\underline{\mathcal{V}}}$ be the unique solutions of $\overline{\delta}$ and $\underline{\delta}$ from $\overline{\omega}$ and $\underline{\omega}$, respectively. We say a pair of dL terms $(\overline{g}, \underline{g}) \in \mathcal{T}(\overline{\mathcal{V}}) \times \mathcal{T}(\underline{\mathcal{V}})$ *synchronizes* $(\overline{\delta}, \underline{\delta})$ from $(\overline{\omega}, \underline{\omega})$ if the following hold.

- $\overline{g}_{\overline{\psi}}(0) = \underline{g_\psi}(0)$
- The derivatives of $\overline{g}_{\overline{\psi}}$ and $\underline{g_\psi}$ are both strictly positive.

The following lemma ensures that, for any synchronizer, a corresponding time stretch function exists.

**Lemma 20.** *In the setting of Definition 19, let $\overline{t} \in [0, \overline{T})$ and $\underline{t} \in [0, \underline{T})$ be such that $\overline{g}_{\overline{\psi}}(\overline{t}) = \underline{g_\psi}(\underline{t})$. Then the function $K$, defined by $K(s) := \underline{g_\psi}^{-1}(\overline{g}_{\overline{\psi}}(s))$, is a time stretch function from $[0, \overline{t}]$ to $[0, \underline{t}]$. Moreover we have $\dot{K}(s) = \frac{\dot{\overline{g}_{\overline{\psi}}}(s)}{\dot{\underline{g_\psi}}(K(s))}$.*

*Proof.* Since $\underline{g_\psi}$ is strictly monotonic on $[0, \underline{t}]$, it has an inverse $\underline{g_\psi}^{-1}$ defined from $\underline{g_\psi}([0, \underline{t}])$ to $[0, \underline{t}]$. By assumption we have $\overline{g}_{\overline{\psi}}(0) = \underline{g_\psi}(0)$, and thus $K(0) = \underline{g_\psi}^{-1}(\overline{g}_{\overline{\psi}}(0)) = \underline{g_\psi}^{-1}(\underline{g_\psi}(0)) = 0$. Also since $\overline{g}_{\overline{\psi}}(\overline{t}) = \underline{g_\psi}(\underline{t})$, we see that $\underline{g_\psi}^{-1}$ is defined from $\overline{g}_{\overline{\psi}}([0, \overline{t}])$ to $[0, \underline{t}]$. Thus $K = \underline{g_\psi}^{-1} \circ \overline{g}_{\overline{\psi}}$ is defined from $[0, \overline{t}]$ to $[0, \underline{t}]$.

$$\begin{aligned}
\dot{K}(s) &= \dot{\overline{g}_{\overline{\psi}}}(s) \cdot \left(\dot{\underline{g_\psi}^{-1}}\right)(\overline{g}_{\overline{\psi}}(s)) && \text{derivative of } K = \underline{g_\psi}^{-1} \circ \overline{g}_{\overline{\psi}} \\
&= \frac{\dot{\overline{g}_{\overline{\psi}}}(s)}{\dot{\underline{g_\psi}}(\underline{g_\psi}^{-1}(\overline{g}_{\overline{\psi}}(s)))} && \text{derivative of } \underline{g_\psi}^{-1} \\
&= \frac{\dot{\overline{g}_{\overline{\psi}}}(s)}{\dot{\underline{g_\psi}}(K(s))}
\end{aligned}$$

whose value is positive by assumptions on the derivatives of $\overline{g}_{\overline{\psi}}$ and $\underline{g_\psi}$. □

We remark that time stretch functions we obtain in Lemma 20 are not necessarily expressible as a dL term, as exemplified by the following example.

**Example 21.** Consider two dynamics $\overline{\delta_F} := (\dot{\overline{x}} = \overline{v}, \dot{\overline{v}} = -\overline{v}^2)$ and $\underline{\delta} := (\dot{\underline{x}} = 1)$. Their solutions $\overline{\psi}, \underline{\psi} : \mathbb{R}_{\geq 0} \to \mathbb{R}^2$ from initial value $x = 0, v = 1$ are

$$\overline{\psi}(s) = \left(\log(1+s), (1+s)^{-1}\right) \qquad \underline{\psi}(s) = (s, 0)$$

Now let $\overline{g} = \overline{x}$ and $\underline{g} = \underline{x}$. Then $\overline{g}_{\overline{\psi}}(s) = \log(1+s)$, $\underline{g_\psi} = \underline{g_\psi}^{-1} = \mathsf{id}$ and thus $K(s) = \underline{g_\psi}^{-1}(\overline{g}_{\overline{\psi}}(s)) = \log(1+s)$. This is not rational and not expressible in dL.

Using the syntactic Lie derivative (Definition 12), we state a sound inference rule that does not need $K$ to be represented explicitly. We note that there is strong support for Lie derivatives in the tool KeYmaera X, as a key syntactic operation behind the differential invariant (DI) rule (Definition 13).

**Definition 22.** Let $\overline{\delta} := \left(\dot{\overline{\mathbf{x}}} = \overline{\mathbf{e}} \,\&\, \overline{Q}\right)$ and $\underline{\delta} := \left(\dot{\underline{\mathbf{x}}} = \underline{\mathbf{e}} \,\&\, \underline{Q}\right)$ be two dynamics and let $(\overline{g}, \underline{g}) \in \mathcal{T}(\overline{\mathcal{V}}) \times \mathcal{T}(\underline{\mathcal{V}})$ (which is supposed to be a synchronizer). We define the *synchronized dynamics* of $(\overline{\delta}, \underline{\delta})$ with respect to $(\overline{g}, \underline{g})$ as follows:

$$\overline{\delta} \otimes_{(\overline{g},\underline{g})} \underline{\delta} \;:=\; \left(\dot{\overline{\mathbf{x}}} = \overline{\mathbf{e}},\; \dot{\underline{\mathbf{x}}} = \frac{\mathcal{L}_{\overline{\delta}}\, \overline{g}}{\mathcal{L}_{\underline{\delta}}\, \underline{g}} \cdot \underline{\mathbf{e}}\right) \,\&\, \left(\overline{Q} \wedge \underline{Q} \wedge \mathcal{L}_{\overline{\delta}}\, \overline{g} > 0 \wedge \mathcal{L}_{\underline{\delta}}\, \underline{g} > 0\right)$$

**Lemma 23.** *Let $(\overline{g}, \underline{g})$ be a synchronizer of $(\overline{\delta}, \underline{\delta})$ from $(\overline{\omega}_0, \underline{\omega}_0)$. The following are equivalent, where the semantical transition relations are from Definition 5.*

1. $(\overline{\omega}_0, \underline{\omega}_0) \dashv\!\![\![\overline{\delta};\, \underline{\delta}]\!]\!\!\rightarrow (\overline{\omega}, \underline{\omega})$ *and* $(\overline{\omega}, \underline{\omega}) \in [\![\overline{g} = \underline{g}]\!]$
2. $(\overline{\omega}_0, \underline{\omega}_0) \dashv\!\![\![\overline{\delta} \otimes_{(\overline{g},\underline{g})} \underline{\delta}]\!]\!\!\rightarrow (\overline{\omega}, \underline{\omega})$

*Proof.* We first prove ($1 \Rightarrow 2$). In the proof of Lemma 20, we can observe that $\dot{\overline{g_{\overline{\psi}}}}(s) = [\![\mathcal{L}_{\overline{\delta}}\, \overline{g}]\!]_{\overline{\psi}(s)}$, and analogously, $\dot{\underline{g_{\underline{\psi}}}}(s) = [\![\mathcal{L}_{\underline{\delta}}\, \underline{g}]\!]_{\underline{\psi}(s)}$. Hence we obtain

$$\dot{K}(s) = \frac{[\![\mathcal{L}_{\overline{\delta}}\, \overline{g}]\!]_{\overline{\psi}(s)}}{[\![\mathcal{L}_{\underline{\delta}}\, \underline{g}]\!]_{\underline{\psi}(K(s))}} = \left[\!\!\left[\frac{\mathcal{L}_{\overline{\delta}}\, \overline{g}}{\mathcal{L}_{\underline{\delta}}\, \underline{g}}\right]\!\!\right]_{\rho(s)} \tag{6}$$

where $\rho : [0, \overline{t}) \to \mathbb{R}^{\overline{\mathcal{V}} \uplus \underline{\mathcal{V}}}$ is defined by $\rho(s) := \left(\overline{\psi}(s), \underline{\psi}(K(s))\right)$.

We note that $K : [0, \overline{t}] \to [0, K(\overline{t})]$ is a time-stretch function, and that $\underline{\psi}$ is a solution of $\dot{\underline{\mathbf{x}}} = \underline{\mathbf{e}}$, that is, $\dot{\underline{\psi}}(u) = [\![\underline{\mathbf{e}}]\!]_{\underline{\psi}(u)}$ whenever $0 \le u < \underline{t} = K(\overline{t})$. Combined with Lemma 17, we obtain

$$\left(\dot{\underline{\psi} \circ K}\right)(s) = \dot{K}(s) \cdot [\![\underline{\mathbf{e}}]\!]_{\underline{\psi}(K(s))} = \dot{K}(s) \cdot [\![\underline{\mathbf{e}}]\!]_{\rho(s)} \qquad \text{whenever } 0 \le s < \overline{t}.$$

Hence, with the fact that $\overline{\psi}$ is a solution of $\dot{\overline{\mathbf{x}}} = \overline{\mathbf{e}}$, we obtain

$$\dot{\rho}(s) = \left(\dot{\overline{\psi}}(s), (\dot{\underline{\psi} \circ K})(s)\right) = \left([\![\overline{\mathbf{e}}]\!]_{\rho(s)},\; \dot{K}(s) \cdot [\![\underline{\mathbf{e}}]\!]_{\rho(s)}\right) = [\![\left(\overline{\mathbf{e}},\; \frac{\mathcal{L}_{\overline{\delta}}\, \overline{g}}{\mathcal{L}_{\underline{\delta}}\, \underline{g}} \cdot \underline{\mathbf{e}}\right)]\!]_{\rho(s)}$$

whenever $0 \le s < \overline{t}$. Here the last equality is from (6). This concludes that $\rho$ is a solution of the dynamics $\overline{\delta} \otimes_{(\overline{g},\underline{g})} \underline{\delta}$. It remains to prove that for all $\tau \in [0, \overline{t}]$, $[\![\overline{Q} \wedge \underline{Q} \wedge \mathcal{L}_{\overline{\delta}}\, \overline{g} > 0 \wedge \mathcal{L}_{\underline{\delta}}\, \underline{g} > 0]\!]_{\rho(\tau)}$ is true. This is an easy consequence of item 1, and the fact that $(\overline{g}, \underline{g})$ is a synchronizer of $(\overline{\delta}, \underline{\delta})$ from $(\overline{\omega}_0, \underline{\omega}_0)$.

For the direction ($2 \Rightarrow 1$), let $(\overline{\xi}, \underline{\xi}) : [0, T) \to \mathbb{R}^{\overline{\mathcal{V}}} \times \mathbb{R}^{\underline{\mathcal{V}}}$ be the unique solution of $\overline{\delta} \otimes_{(\overline{g},\underline{g})} \underline{\delta}$ from $(\overline{\omega}_0, \underline{\omega}_0)$. Then there is $t \in [0, T)$ such that $(\overline{\xi}(t), \underline{\xi}(t)) = (\overline{\omega}, \underline{\omega})$. Let us prove that $(\overline{\omega}, \underline{\omega}) \in [\![\overline{g} = \underline{g}]\!]$. The function $h : s \in [0, T) \mapsto [\![\overline{g}]\!]_{\overline{\xi}(s)} - [\![\underline{g}]\!]_{\underline{\xi}(s)}$ is equal to 0 at $s = 0$ and its derivative is given by:

$$\dot{h}(s) = [\![\mathcal{L}_{\overline{\delta}}\, \overline{g}]\!]_{\overline{\xi}(s)} - [\![\mathcal{L}_{\underline{\delta}}\, \underline{g}]\!]_{\underline{\xi}(s)} \cdot \frac{[\![\mathcal{L}_{\overline{\delta}}\, \overline{g}]\!]_{\overline{\xi}(s)}}{[\![\mathcal{L}_{\underline{\delta}}\, \underline{g}]\!]_{\underline{\xi}(s)}} = 0$$

Consequently, $h$ is the constant function equal to 0, which implies that $(\overline{\omega}, \underline{\omega}) \in [\![\overline{g} = \underline{g}]\!]$. By definition, $\overline{\xi}$ is a solution of $\overline{\delta}$, so $\overline{\omega}_0 \dashv\![\![\overline{\delta}]\!]\!\!\rightarrow \overline{\omega}$. Furthermore, by Corollary 18, $\underline{\xi} \circ K^{-1}$ is a solution of $\underline{\delta}$. Thus $\underline{\omega}_0 \dashv\![\![\underline{\delta}]\!]\!\!\rightarrow \underline{\omega}$ and

$$(\overline{\omega}_0, \underline{\omega}_0) \dashv\![\![\overline{\delta};\ \underline{\delta}]\!]\!\!\rightarrow (\overline{\omega}, \underline{\omega}). \qquad \square$$

The above lemma is a key observation in the current work. It allows us to turn the relational dynamics $\overline{\delta}; \underline{\delta}$—expressed as a sequential composition in dL—into a combined dynamics $\overline{\delta} \otimes_{(\overline{g}, \underline{g})} \underline{\delta}$. Moreover, we can do so in a way that the two dynamics are synchronized in a reparametrized manner, as specified by $(\overline{g}, \underline{g})$. Such combination of two dynamics is crucial in exploiting the logical infrastructure of dL and KeYmaera X—we emphasize again that the (DI) rule does not support invariant reasoning about the relationship between $\overline{\delta}$ and $\underline{\delta}$, when the relational dynamics is expressed in the original form $\overline{\delta};\ \underline{\delta}$.

The following is an incarnation of Lemma 23 as a proof rule. We assume that a postcondition is a conditional form $E \Rightarrow \varphi$; $E$ is called an *exit condition*. By assuming that $E$ implies $\overline{g} = \underline{g}$, we enforce the second condition $(\overline{\omega}, \underline{\omega}) \in [\![\overline{g} = \underline{g}]\!]$ in item 1 of Lemma 23. The first three premises are there to ensure that $(\overline{g}, \underline{g})$ is a synchronizer. Under these premises (the first four), the rule allows one to transform its conclusion (about $\overline{\delta}; \underline{\delta}$) into one about the combined dynamics $\overline{\delta} \otimes_{(\overline{g}, \underline{g})} \underline{\delta}$, which is amenable to application of the (DI) rule, for example.

**Theorem 24 (synchronization rule).** *The following inference rule is sound:*

$$\frac{\begin{array}{l} \Gamma \vdash [\,\overline{\delta}\,]\mathcal{L}_{\overline{\delta}}\overline{g} > 0 \quad \Gamma \vdash \overline{g} = \underline{g} \\ \Gamma \vdash [\,\underline{\delta}\,]\mathcal{L}_{\underline{\delta}}\underline{g} > 0 \quad E \vdash \overline{g} = \underline{g} \quad \Gamma \vdash [\,\overline{\delta} \otimes_{(\overline{g}, \underline{g})} \underline{\delta}\,](E \Rightarrow \varphi) \end{array}}{\Gamma \vdash [\,\overline{\delta}; \underline{\delta}\,](E \Rightarrow \varphi)}\ (Sync)$$

Recall the definition of $\overline{\delta} \otimes_{(\overline{g}, \underline{g})} \underline{\delta}$ (Definition 22), where time stretching for the second dynamics $\underline{\delta}$ is expressed syntactically by Lie derivatives. We call the four premises $\Gamma \vdash \overline{g} = \underline{g}$, $E \vdash \overline{g} = \underline{g}$, $\Gamma \vdash [\,\overline{\delta}\,]\mathcal{L}_{\overline{\delta}}\overline{g} > 0$, and $\Gamma \vdash [\,\underline{\delta}\,]\mathcal{L}_{\underline{\delta}}\underline{g} > 0$ the *synchronizability conditions*. These obligations are usually easy to discharge. The last premise, which we call the *synchronized formula*, is typically the core remaining obligation.

**Remark 25 (choice of $(\overline{g}, \underline{g})$).** In applying the (Sync) rule, one still has to find a suitable synchronizer $(\overline{g}, \underline{g})$. This turns out to be straightforward in many examples. In all the case studies in Section 6 and in Example 1, the exit condition $E$ is of the form $\overline{x} = \underline{x} = C$ where $C$ is a constant. This suggests the use of $\overline{g} = \overline{x}$, $\underline{g} = \underline{x}$. Indeed, all our proofs use this choice of $(\overline{g}, \underline{g})$.

### 5 Implementation

KeYmaera X [17] is an interactive theorem prover based on the sequent calculus formulation of dL. It is implemented in Scala, replacing its former system KeYmaera [16]. It has a web-based GUI environment, and a support of automated theorem proving using computer algebra systems such as Mathematica [27].

For the formalization of case studies in Section 6, we extended KeYmaera X version 4.7 (available at [17]) with the (Sync) rule. This extension of KeYmaera X, together with our proofs in case studies, are currently available at http://group-mmm.org/rddl_tacas_2020/.

The KeYmaera X implementation is structured in a flexible manner, from which we benefited. To add a rule to KeYmaera X, one has to implement a Scala program that take the conclusion of the rule and generate the premises of the rule as subgoals. The fact that any Scala program is allowed here enabled us to implement complex algorithms, such as inductive translation of formulas.

In implementing the (Sync) rule, the functions in KeYmaera X called *helpers* helped us, such as in the Lie derivative computation and the functionality to simplify formulas into equivalent ones. The bulk of our effort regarded the $\otimes_{(\overline{g},\underline{g})}$ operator. There we did a bit more general than we stated in the paper: not only taking dynamics of form $\dot{\mathbf{x}} = \mathbf{e} \,\&\, Q$, we also allow sequences of dynamics possibly interleaved by guards and nondeterministic choices. This feature was utilized in the case study that will be described in Section 6.3.

## 6 Case Studies

We describe three case studies where we proved relational properties of hybrid dynamics. We did so formally in our extension of KeYmaera X described in Section 5. In all the examples, we apply the (Sync) rule as a main proof step, in conjunction with the existing rules in dL. Below, we describe our example systems and outline the important steps in the formal proofs.

### 6.1 Collision Speed with Constant Acceleration

In this section we apply the (Sync) rule to the running Example 1. For this example we consider two dynamics $\overline{\delta_C} := \left(\dot{\overline{x}} = \overline{v}, \dot{\overline{v}} = \overline{a}\right)$ and $\underline{\delta_C} := \left(\dot{\underline{x}} = \underline{v}, \dot{\underline{v}} = \underline{a}\right)$. Both dynamics represent a car with constant acceleration. Our claim is that if acceleration is larger in the first system, then the first car is necessarily faster than the second car after traveling the same distance $l$; formally,

$$\Gamma \vdash \left[\overline{\delta_C}; \underline{\delta_C}\right](\overline{x} = l \wedge \underline{x} = l \Rightarrow \underline{v} \leq \overline{v}) \tag{7}$$

where

$$\Gamma := \{0 = \overline{x} = \underline{x},\ \ 0 < \overline{v} = \overline{v}_0,\ \ \underline{v} = \underline{v}_0,\ \ \overline{v}_0 \geq \underline{v}_0,\ \ 0 \leq \underline{a} \leq \overline{a}\}$$

We apply the (Sync) rule, where $\overline{g} := \overline{x}$ and $\underline{g} := \underline{x}$. The first two synchronizability conditions are $\Gamma \vdash \underline{x} = \overline{x}$ and $\underline{x} = l,\ \overline{x} = l \vdash \underline{x} = \overline{x}$, which are trivial. The last two synchronizability conditions are

$$\Gamma \vdash \left[\overline{\delta_C}\right]\mathcal{L}_{\overline{\delta_C}}\,\overline{g} = \overline{v} > 0 \qquad\qquad \Gamma \vdash \left[\underline{\delta_C}\right]\mathcal{L}_{\underline{\delta_C}}\,\underline{g} = \underline{v} > 0$$

which are proven using differential invariants (DI). The synchronized formula is

$$\Gamma \vdash \left[\overline{\delta_C}, \dot{\underline{x}} = \underline{v} \cdot (\overline{v}/\underline{v}), \dot{\underline{v}} = \underline{a} \cdot (\overline{v}/\underline{v}) \,\&\, \overline{v} > 0 \wedge \underline{v} > 0\right](\overline{x} = l \wedge \underline{x} = l \Rightarrow \underline{v} \leq \overline{v})$$

One might try to show the inequality $\overline{v} - \underline{v} \geq 0$ by the differential invariant (DI) rule, but the Lie derivative of the term $\overline{v} - \underline{v}$ is $\overline{a} - \underline{a} \cdot (\overline{v}/\underline{v})$, which is not obviously nonnegative. Instead, a trickier expression $\overline{a} \cdot (\underline{v}^2 - \underline{v}_0^2) - \underline{a} \cdot (\overline{v}^2 - \overline{v}_0^2) = 0$ turns out to be an invariant. Its Lie derivative is $\overline{a} \cdot (2\underline{v}) \cdot \underline{a} \cdot (\overline{v}/\underline{v}) - \underline{a} \cdot (2\overline{v}) \cdot \overline{a}$, which is clearly 0, since we also know $\underline{v} > 0$.

We do not have an intuitive explanation for this invariant, but it was found by a template-based search, like many other invariants in dL. By positing the existence of a polynomial invariant of a certain degree, we can find conditions on the coefficients by requiring its Lie derivative and initial value are zero. Solving these conditions for a second-degree invariant on the velocities in the system yielded the invariant above.

After finding our invariant, we additionally have to show the invariant entails our desired result, $\underline{v} \leq \overline{v}$. This can be shown with a standard monotonicity property of modal logics: from $\phi \vdash \psi$ and $\Gamma \vdash [\alpha]\phi$, we can conclude $\Gamma \vdash [\alpha]\psi$, where $\phi$ states the expression above is an invariant and the velocities are always greater than their initial value, and $\psi$ is our goal: $\underline{v} \leq \overline{v}$.

#### 6.2 Collision Speed with Different Kinds of Friction

Here we continue Example 11, where we consider two dynamics $\overline{\delta_F} \equiv (\dot{\overline{x}} = \overline{v}, \dot{\overline{v}} = -\overline{v}^2)$ and $\underline{\delta_F} \equiv (\dot{\underline{x}} = \underline{v}, \dot{\underline{v}} = -\underline{v})$. Our goal is $\Gamma_F \vdash [\overline{\delta_F}; \underline{\delta_F}](\overline{x} = \underline{x} = l \Rightarrow \underline{v} \leq \overline{v})$, with $\Gamma_F := \{\overline{x} = \underline{x} = 0,\ 0 < \underline{v} \leq \overline{v} \leq 1\}$.

First, we establish the fact that the objects in this example always have positive velocity. We show this by the (Dbx) rule (Definition 13), where $\mathcal{L}_{\overline{\delta_F}} \overline{v} = -\overline{v}^2$ and $\mathcal{L}_{\underline{\delta_F}} \underline{v} = -\underline{v}$. This allows us to infer $\overline{v} > 0$ and $\underline{v} > 0$ hold at all times.

We apply the (Sync) rule along $\overline{x} = \underline{x}$, yielding the synchronized dynamics

$$\dot{\overline{x}} = \overline{v},\ \dot{\overline{v}} = -\overline{v}^2,\ \dot{\underline{x}} = \underline{v} \cdot (\overline{v}/\underline{v}),\ \dot{\underline{v}} = -\underline{v} \cdot (\overline{v}/\underline{v}) \ \ \&\ \ \overline{v} > 0 \wedge \underline{v} > 0$$

Note that the new evolution domain condition $\underline{v} > 0$ allows us to rewrite $\underline{v} \cdot (\overline{v}/\underline{v})$ to $\overline{v}$. The synchronizability conditions follow immediately from the fact that $\underline{v} > 0$ and $\overline{v} > 0$. For the synchronized formula, we apply the (DI) rule, so the desired inequality $\overline{v} \geq \underline{v}$ is reduced to $\overline{v}^2 \leq \overline{v}$, that is, $\overline{v} \leq 1$. To this end, $\overline{v} > 0$ tells us that the derivative of $\overline{v}$, that is, $-\overline{v}^2$, is always negative, therefore $\overline{v} \leq 1$.

#### 6.3 Model Refinement

In this example, we consider two abstract models of cars. The first car is able to provide a high amount of constant acceleration $a$ at low velocities, but at a certain velocity $v_{cut}$ the engine switches to a different mode and then provides a lesser, but still constant acceleration $a_{cut}$. The second car is an abstracted version of the first, which ignores this mode change and provides the same constant amount of acceleration $a$ at all velocities. Our aim in this example is to establish a safety envelope around the first car's behavior using the more simply stated second car's dynamics. Hence we show that the second car's velocity is greater than the first's at any position $\overline{x} = \underline{x} = l$. More formally, the behavior of the first car is expressed as a hybrid program $\overline{\alpha} := (\overline{\delta_1};\ ?\overline{v} = v_{cut};\ \overline{\delta_2})$ with two modes: $\overline{\delta_1} := (\dot{\overline{x}} = \overline{v}, \dot{\overline{v}} = a \,\&\, \overline{v} \leq v_{cut})$ and $\overline{\delta_2} := (\dot{\overline{x}} = \overline{v}, \dot{\overline{v}} = a_{cut})$. The second car follows the simple dynamics $\underline{\delta} := (\dot{\underline{x}} = \underline{v}, \dot{\underline{v}} = a)$. Our goal is to prove the sequent $\Gamma \vdash [\overline{\alpha}; \underline{\delta}](\underline{x} = \overline{x} = l \Rightarrow \underline{v} \leq \overline{v})$, where the initial conditions are given by

$$\Gamma := (\overline{x} = \underline{x} = 0, 0 < \overline{v} = \underline{v} = v_0, 0 < v_{cut}, 0 < a_{cut} \leq a)$$

Technically, the (Sync) rule merges one differential dynamics with another, but the program the first car executes is a more complicated composition of dynamics and testing. However, it is possible to synchronize *piecewise*, first synchronizing $\underline{\delta}$ with $\overline{\delta_1}$ until the first car changes modes, then synchronizing $\underline{\delta}$ with $\overline{\delta_2}$ for the remainder of their runs. This slightly generalized synchronization procedure means that we can instead show

$$\Gamma \vdash \left[\overline{\delta_1} \otimes_{(\overline{x},\underline{x})} \underline{\delta}; ?\overline{v} = v_{cut}; \overline{\delta_2} \otimes_{(\overline{x},\underline{x})} \underline{\delta}\right](\underline{x} = \overline{x} = l \Rightarrow \underline{v} \leq \overline{v})$$

There are also now two sets of synchronizability conditions to satisfy, but both are again straightforward. Since $\overline{\delta_1}$ and $\underline{\delta}$ are nearly identical (except for the evolution domain constraint), their synchronization $\overline{\delta_1} \otimes_{(\overline{x},\underline{x})} \underline{\delta}$ basically identifies the two dynamics. The synchronization of $\overline{\delta_2}$ and $\underline{\delta}$ is exactly the synchronization performed above in Section 6.1, and proceeds in the same way.

### 7 Conclusions and Future Work

In this paper, we present a relational extension of the differential dynamic logic based on time stretching of dynamics. This reparametrization enables us to enforce that comparisons between two systems occur when certain conditions are satisfied, for example when two cars are passing through the same position. While such reparametrizations can be thought of as stretching or compressing time for one of the dynamics, we also show they can be conducted by a transformation of the dynamics themselves, based on Lie derivatives. We call this process *synchronizing* the dynamics (Definition 19), and it leads us to a new dL proof rule, the (Sync) rule (Theorem 24). We implemented the new rule in the KeYmaera X tool and use our extension to demonstrate several nontrivial relational properties of dynamical systems.

In the future, we think it would be interesting to combine our relational logic with orthogonal relational extensions of dL [14] which focus on *refinement relations* with varying levels of nondeterminism. We also hinted in our last case study that it is possible to synchronize wider classes of hybrid programs than just two differential dynamics. We also think that the level of automated proof search available in KeYmaera X may enable the automatic detection of monotonic properties in *product lines*. This may be useful in industry both to provide sanity checks on formalized models of products, as well as enabling strong guarantees to be more easily obtained for those models.
